# Orally Administered P22 Phage Tailspike Protein Reduces *Salmonella* Colonization in Chickens: Prospects of a Novel Therapy against Bacterial Infections

**DOI:** 10.1371/journal.pone.0013904

**Published:** 2010-11-22

**Authors:** Shakeeba Waseh, Pejman Hanifi-Moghaddam, Russell Coleman, Michael Masotti, Shannon Ryan, Mary Foss, Roger MacKenzie, Matthew Henry, Christine M. Szymanski, Jamshid Tanha

**Affiliations:** 1 Institute for Biological Sciences, National Research Council of Canada, Ottawa, Canada; 2 Department of Environmental Biology, Ontario Agricultural College, University of Guelph, Guelph, Canada; 3 Department of Discovery Research, Dow AgroSciences, Indianapolis, Indiana, United States of America; 4 Department of Biochemistry, Microbiology and Immunology, University of Ottawa, Ottawa, Canada; Cairo University, Egypt

## Abstract

One of the major causes of morbidity and mortality in man and economically important animals is bacterial infections of the gastrointestinal (GI) tract. The emergence of difficult-to-treat infections, primarily caused by antibiotic resistant bacteria, demands for alternatives to antibiotic therapy. Currently, one of the emerging therapeutic alternatives is the use of lytic bacteriophages. In an effort to exploit the target specificity and therapeutic potential of bacteriophages, we examined the utility of bacteriophage tailspike proteins (Tsps). Among the best-characterized Tsps is that from the *Podoviridae* P22 bacteriophage, which recognizes the lipopolysaccharides of *Salmonella enterica* serovar Typhimurium. In this study, we utilized a truncated, functionally equivalent version of the P22 tailspike protein, P22sTsp, as a prototype to demonstrate the therapeutic potential of Tsps in the GI tract of chickens. Bacterial agglutination assays showed that P22sTsp was capable of agglutinating *S.* Typhimurium at levels similar to antibodies and incubating the Tsp with chicken GI fluids showed no proteolytic activity against the Tsp. Testing P22sTsp against the three major GI proteases showed that P22sTsp was resistant to trypsin and partially to chymotrypsin, but sensitive to pepsin. However, in formulated form for oral administration, P22sTsp was resistant to all three proteases. When administered orally to chickens, P22sTsp significantly reduced *Salmonella* colonization in the gut and its further penetration into internal organs. In *in vitro* assays, P22sTsp effectively retarded *Salmonella* motility, a factor implicated in bacterial colonization and invasion, suggesting that the *in vivo* decolonization ability of P22sTsp may, at least in part, be due to its ability to interfere with motility… Our findings show promise in terms of opening novel Tsp-based oral therapeutic approaches against bacterial infections in production animals and potentially in humans.

## Introduction

Bacterial infections are one of the major causes of morbidity and mortality in both man and economically important animals and impose a huge economical burden in terms of health care cost, lost manpower and contaminated food. Antibiotics are widely used and in some places are the only treatments available. However, due to the emergence of difficult-to-treat infections caused by antibiotic resistant bacteria, there is an urgent need for alternatives. Several other therapeutic approaches involving passive and active immunization, toxin binding agents, lytic bacteriophages, anti-bacterial peptides, phage lytic enzymes and probiotics have been used but they are far from replacing the antibiotic therapy approach [1]–[9].

Lytic bacteriophages are the natural enemy of their host bacteria and, as such, provide a natural solution for the therapy of bacterial infections. Bacteriophages are also host specific, self-replicating, self limiting and virtually non-toxic. These characteristics have made lytic bacteriophages attractive therapeutic agents to combat bacterial infections. Despite their widely known therapeutic potential, bacteriophages have still not filled the gap of desperately needed alternatives to current antibacterial agents. Antibacterial activities of bacteriophages have been tested in several animal studies but led to, often, mixed outcomes [10]–[15]. Bacteriophage therapy is associated with drawbacks such as the possibility of showing reduced efficacy under anaerobic conditions in the gut due to bacterial regrowth [16], the emergence of phage-resistant bacteria and the risk of horizontal gene transfer of virulence traits to the bacteria potentially rendering the target organism even more pathogenic [17]–[19].

To exploit the target specificity and therapeutic potential of bacteriophages without being held back by some of the drawbacks associated with using whole phages, we chose to investigate the utility of bacteriophage tailspike proteins (Tsps). Tsps are components of the tail apparatus of many bacteriophages, and mediate the specific recognition of its bacterial host by binding to surface structures such as polysaccharides [20]–[27]. Additionally, many Tsps have endoglycosidase activity, hydrolyzing their polysaccharide receptors. Tsps identified to date are homotrimers consisting of an N-terminal capsid-binding domain, a central domain that binds/hydrolyzes the O-antigen region of bacterial surface lipopolysaccharides and a C-terminal region crucial for trimerization. A hallmark of Tsps is their high stability. Tsps, as well as their N-terminal truncated versions, are protease resistance, have excellent thermostability, show resistance to dissociation in high concentrations of urea and SDS, and reversibly unfold in concentrated chemical denaturants [23], [25], [26]–[29]. One well studied Tsp is that of bacteriophage P22 which is a 215-kDa trimeric protein specifically recognizing several pathogenic *Salmonella* spp. including *S. enterica* serovar Typhimurium [30]–[33]. The shortened version of P22 phage Tsp (P22sTsp) lacking the N-terminal capsid-binding domain exhibits the same native structure, oligosaccharide binding and endorhamnosidase activity as the full-length Tsp [28], [29], [33]. Similar to the full-length Tsp, the shortened P22 Tsp exhibits impressive solubility and stability properties: it is SDS- and trypsin-resistant as well as thermostable at temperatures beyond 80°C [28], [29], [34], [35].

Here, we tested the idea of using bacteriophage Tsps as an alternative to the whole phage approach for oral therapy of bacterial gut infections. The general stability of Tsps and their resistance to proteases makes Tsps - as well as their shortened derivatives - ideal proteins for oral therapeutics as they can better resist the acid-induced denaturation and digestion by proteases in the gastrointestinal (GI) tract. In this study we show that the *Salmonella*-specific P22sTsp is effective in reducing *Salmonella* colonization in the gut of chickens and the penetration into internal organs. Our findings show promise in terms of opening novel oral Tsp-based therapeutic approaches against bacterial infections in both man and production animals that provide many advantages over the use of intact bacteriophages including reduced ability of the pathogen to develop resistance and release of harmful bacterial cell components during lysis.

## Results

### P22sTsp agglutinates *Salmonella* cells similar to antibodies

**Figure 1:**
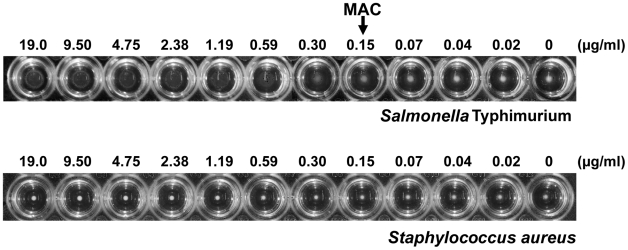
P22sTsp micro-agglutination assay at 4°C. P22sTsp agglutinates *Salmonella* effectively as shown by the diffused cell patterns (wells on the left side of the arrow). No agglutination was observed with *Staphylococcus aureus* (lower panel) at the highest concentration used (cell sediments appear as round dots). MAC: minimum agglutination concentration.

The ability of P22sTsp to agglutinate *Salmonella* cells was assessed in cell micro-agglutination assays. Because P22sTsp is multimeric, it is expected to cross-link and agglutinate the bacterial cells. In micro-agglutination assays, bacteria that are not agglutinated settle to the center of the well forming a dot and those that are agglutinated form a sheet across the well [36] (Figure 1). Micro-agglutination was performed by incubating a constant number of bacterial cells with two-fold dilutions of P22sTsp.

The agglutination ability of P22sTsp was assessed at 4°C and 42°C. At 4°C, the minimum concentration of P22sTsp resulting in detectable cell agglutination (minimum agglutination concentration) was 149 ng/ml (Figure 1). P22sTsp showed slightly better agglutination than the Se155-4 antibody [37], which is specific to the O-antigen of *Salmonella enterica* serovar Typhimurium (minimum agglutination concentration  = 320 ng/ml). The agglutination was specific since P22sTsp did not agglutinate the control organism, *Staphylococcus aureus* (Figure 1). No agglutination was observed at 42°C (physiological body temperature in chickens) under the examined assay conditions. This can be attributed to the endorhamnosidase activity of P22sTsp interfering with its binding to *Salmonella* and subsequent agglutination, an activity which is presumably suppressed at 4°C. Consistent with this view, a P22sTsp mutant (D392N; [33]) with wild-type binding affinity and defective endorhamnosidase activity agglutinates *Salmonella* at 42°C (unpublished results).

### P22sTsp is significantly resistant to GI proteases

**Figure 2:**
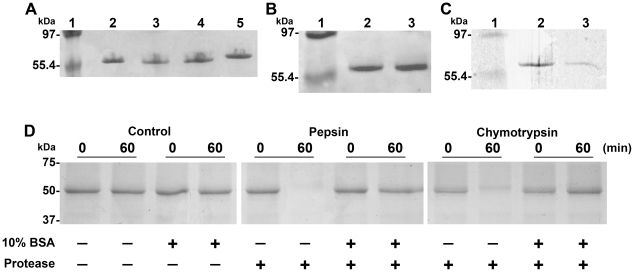
Protease resistance profile of P22sTsp. SDS-PAGE analysis of P22sTsp following treatment with **A**, chicken GI fluid proteases, **B**, trypsin, **C**, chymotrypsin and **D,** pepsin and chymotrypsin in the presence or absence of 10% BSA. **A,** Lane 1, molecular weight markers; lane 2, untreated P22sTsp; lanes 3–5, P22sTsp incubated with protease solutions at 37°C for 5 min, 20 min and 2 h, respectively. **B**, **C**, Lane 1, molecular weight marker; lane 2, untreated P22sTsp; lane 3, trypsin-treated (**B**) or chymotrypsin-treated (**C**) P22sTsp (1 h at 37°C). A control protein (single-domain antibody) incubated with the GI fluid (37°C, 2 h), trypsin or chymotrypsin (37°C, 1 h) showed complete digestion (data not shown). **D**, All reaction samples, including controls (no proteases) were subjected to a purification step with PureProteome™ Nickel Magnetic Beads prior to their analysis by SDS-PAGE (see Materials and Methods). Digestion reactions were carried out for 0 and 60 min. The positions of the molecular weight markers are indicated on the left of the panel D figures.

In the present study, P22sTsp was fed to chickens in order to assess its ability to reduce *Salmonella* colonization in the gut. The decolonizing efficacy of P22sTsp would depend on its degree of resistance to GI proteases, particularly to trypsin, chymotrypsin and pepsin, the major GI proteases. We, thus, performed a series of GI protease digestion experiments to obtain the resistance profile of P22sTsp. In the first set of experiments, susceptibility of P22sTsp to proteases present in chicken GI fluids was tested. P22sTsp was shown to be completely resistant to GI fluid proteases even after 2 h of incubation at 37°C, whereas a control antibody protein was completely digested (Figure 2A). Also, the antibody control was not digested when incubated with heat-inactivated GI fluid for 2 h at 37°C, demonstrating that the banding pattern was due to proteases in the GI fluids (data not shown). The P22sTsp was also shown to be completely resistant to trypsin (Figure 2B), but somewhat sensitive to chymotrypsin (Figure 2C) and completely sensitive to pepsin digestion (data not shown). However, when 10% bovine serum albumin (BSA) was included in the digestion reactions, chymotrypsin and pepsin did not digest P22sTsp anymore, demonstrating the protective role of BSA against these proteases (Figure 2D). Thus, to diminish the possible adverse effects of variable GI proteases *in vivo*, the following formulation was chosen for the animal studies: P22sTsp suspended in PBS (pH 7) containing 10% BSA.

### P22sTsp reduces *Salmonella* colonization in the gut and bacterial penetration into internal organs

Next, we set out to investigate, in a series of animal studies, the effect of oral administration of P22sTsp in reducing *Salmonella* colonization in chicks. Subsequent to *Salmonella* colonization of the GI tract, the organism has been shown to translocate through the gut into the bloodstream, leading to substantial infection of the liver and spleen [38]–[41]. Thus, the effect of treatment was assessed by counting the number of viable *Salmonella* not only in the gut (cecum) but also in the liver and spleen.

**Figure 3:**
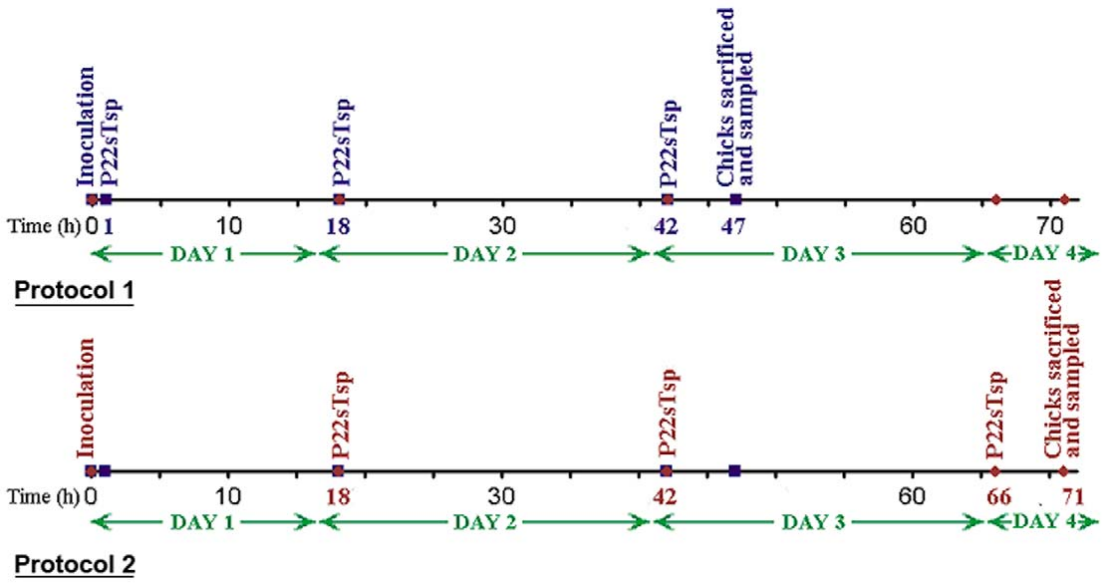
Schematic overview of the two protocols used for animal studies. At time point zero, chicks were inoculated with 10^7^ *Salmonella*. In Protocol 1, chicks were gavaged after inoculation (1 h) with P22sTsp in 10% BSA or with 10% BSA alone. The next two gavages were given at 18 h and 42 h. Chicks were sacrificed at 47 h. In Protocol 2, the first gavage was delayed by 17 h and given at 18 h. The next two gavages were given at 42 h and 66 h. The chicks were subsequently sacrificed at 47 h (Protocol 1) or 71 h (Protocol 2).

For the animal studies, two-day-old chicks were orally infected with *Salmonella* (10^4^ to 10^7^ colony-forming units (CFU), depending on the experiment) and were subsequently fed with 3 doses of P22sTsp (in PBS/10% BSA). In preliminary experiments two different treatment protocols were tested (Protocol 1 and Protocol 2, Figure 3). The first dose of Tsp in Protocol 1 was given one hour after infection of chicks with *Salmonella* (10^7^ CFU) and in Protocol 2, the dose was delayed and given at time 18 h post infection. Following the first dose, the second and third doses were given in 24-hour time intervals in both protocols. Chicks were then sacrificed 5 h after receiving the third dose and their *Salmonella* contents were enumerated in the cecum, liver and spleen.

**Figure 4:**
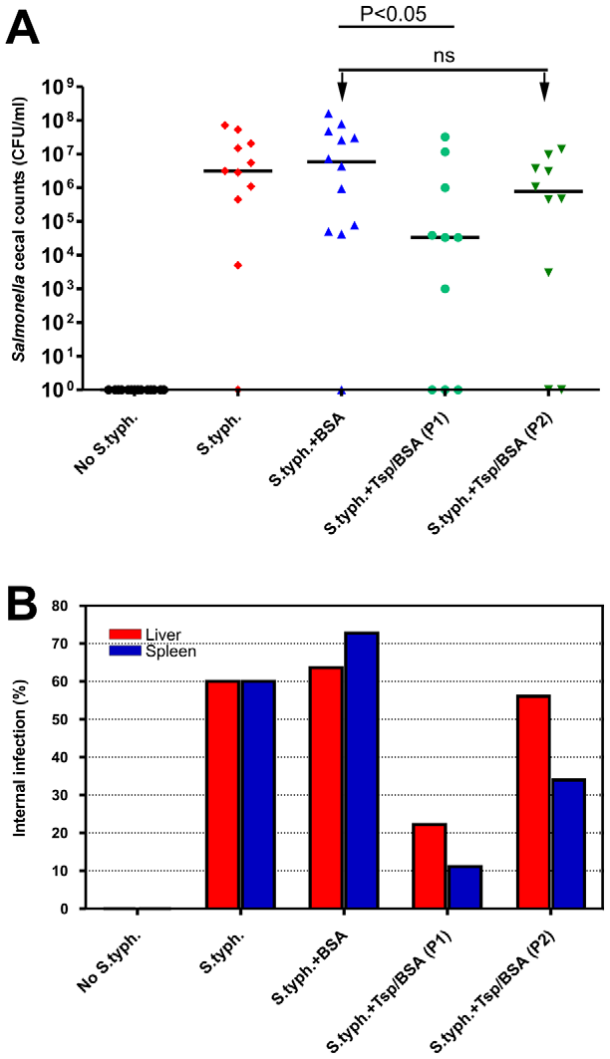
Effect of orally administered P22sTsp on *Salmonella* colonization of chick cecum. Effect of orally administered P22sTsp on *Salmonella* colonization of chick cecum (A) or liver and spleen (B). Medians are shown as horizontal bars on graph A. The two protocols were compared and for subsequent studies Protocol 1 was used. Chicks not infected with *Salmonella* Typhimurium (No S.typh.); chicks infected with *S.* Typhimurium but did not receive any treatment (S.typh.); infected chicks treated with 10% BSA (S.typh.+BSA); infected chicks treated with P22sTsp in 10% BSA according to Protocol 1 (S.typh.+Tsp/BSA (P1)); infected chicks treated with P22sTsp in 10% BSA according to Protocol 2 (S.typh.+Tsp/BSA (P2)). For cecal results, the overall treatment effect was assessed using the Kruskal-Wallis test with Dunnett's post test to correct for the number of comparisons and was significant (P<0.05). For liver and spleen results, all pairs were compared to each other using the chi square test. Not significant (ns).

P22sTsp treatment of infected animals led to a significant reduction of *Salmonella* in the cecum, liver and spleen in Protocol 1 (P<0.05) but not in Protocol 2 (Figures 4A and 4B). Infected animals receiving Tsp treatments under Protocol 1 (S.typh.+Tsp/BSA (P1)) and Protocol 2 (S.typh.+Tsp/BSA (P2)) showed at least 100- and 10-fold reduction of bacterial titre in their ceca, respectively (P1 median = 3.3×10^4^ CFU/ml; P2 median = 7.8×10^5^ CFU/ml), compared to control groups which were infected with *Salmonella* and either treated with BSA alone (S.typh.+BSA) or not treated (S.typh.) (medians: 5.9×10^6^ CFU/ml and 3.2×10^6^ CFU/ml, respectively, Figure 4A).

Examination of bacterial infection in the liver and spleen showed the same overall pattern as those for the cecum (Figures 4A and 4B). *Salmonella* was detected in the livers and spleens of 65–73% of the control group that was treated with BSA alone (S.typh.+BSA) and 60% of the non-treated control group (S.typh.). Percentages of livers infected were significantly lower for animals that were treated according to Protocol 1 (P<0.0001) but not Protocol 2 compared to the BSA-treated groups (Table 1). However, both protocols were effective in reducing the infection in the spleens of Tsp-treated animals compared to the BSA-treated controls (P<0.0001), with Protocol 1 being more effective than Protocol 2 (P<0.0001; Table 1).

**Table 1:** Comparative study of two different treatment protocols.

	Infected/not infected			P values (chi square)		
	Cont.(+/−)	P1(+/−)	P2(+/−)	Cont. vs P1	Cont. vs P2	P1 vs P2
Liver	64/36	22/78	56/44	P<0.0001	ns	P<0.0001
Spleen	73/27	11/89	33/67	P<0.0001	P<0.0001	P<0.0001

Protocol 1 (P1); Protocol 2 (P2); Control (Cont.); Not significant (ns); Percentage infected with *Salmonella* versus not infected (+/−). The Chi square test was used to compare pairs. P values less than 0.05 were considered significant.

**Figure 5:**
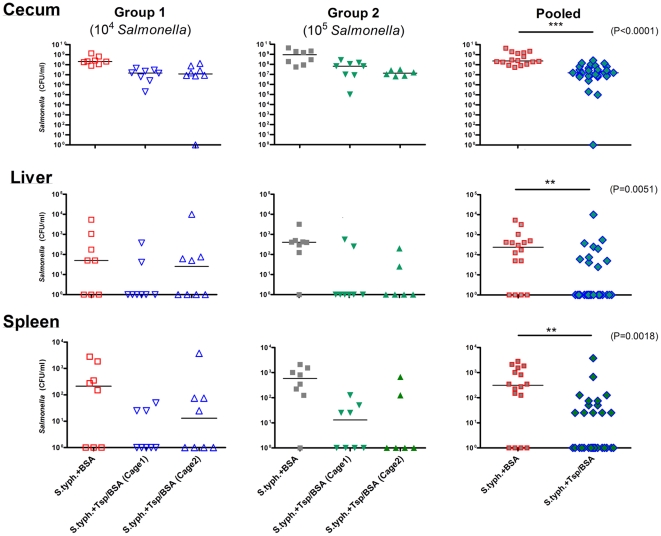
Effect of orally administered P22sTsp on *Salmonella* colonization of chick cecum, liver and spleen at an inoculation level of 10^4^ (Group 1) and 10^5^ (Group 2) bacteria. Medians are shown as horizontal bars on the graphs. Protocol 1 was followed. The P22sTsp treatment in each group was done in duplicate (cage 1 and cage 2). Non-inoculated chicks remained pathogen free (n = 9) and are not included in the graph. Infected chicks treated with 10% BSA (S.typh.+BSA); infected chicks treated with P22sTsp in 10% BSA (S.typh.+Tsp/BSA). The data from S.typh.+BSA cohorts of group 1 and group 2 were pooled, since there was no significant difference between the two groups (Mann Whitney two-tailed t-test, cecum: P = 0.32; liver: P = 0.23; spleen: P = 0.5). Pooled data for Tsp-treated animals includes 4 cohorts (2 cages, two groups). For comparison within the groups see Table 2.

Protocol 1 was then used in all subsequent trials. Two-day-old chicks were divided into two groups, each consisting of 3 cages with 8 chicks (total of 24 per group, Figure 5). Following inoculation with either 10^4^ bacteria (Group 1) or 10^5^ bacteria (Group 2), one cage in each group received BSA (S.typh.+BSA) while the other two received P22sTsp (S.typh.+Tsp/BSA). Ceca of Tsp-treated chicks (S.typh.+Tsp/BSA), from groups 1 and 2, showed a significant reduction of *Salmonella* counts compared to the BSA-treated cohort (Group 1, p = 0.0007; Group 2, p = 0.003, Figure 5, Table 2). The reduction was 13- and 15-fold for group 1 (medians: 1.5×10^7^ CFU/ml and 1.2×10^7^ CFU/ml, cage 1 and cage 2, respectively) and 13- and 70-fold for group 2 (medians: 6.6×10^7^ CFU/ml and 1.3×10^7^ CFU/ml, cage 1 and cage 2, respectively) when compared to the BSA-treated animals (medians: 2.0×10^8^ CFU/ml and 9.1×10^8^ CFU/ml; group 1 and group 2, respectively). Pooled data from group 1 and group 2 of Tsp-treated animals were compared to pooled data from BSA-treated animals and showed a significant reduction in *Salmonella* counts in the cecum, liver and spleen (Figure 5, Table 2).

**Table 2:** Effect of P22sTsp on reducing *Salmonella* colonization in the cecum, liver and spleen of two-day-old chicks.

Group 1	All cohorts^1^	Cage 1 vs Cont.	Cage 2 vs Cont.	Cage 1 vsCage 2
Cecum	P<0.001	P<0.01	P<0.01	ns
Liver	ns	ns	ns	ns
Spleen	ns	ns	ns	ns

Group 2				
Cecum	P<0.01	ns	P<0.01	ns
Liver	P<0.05	P<0.05	P<0.05	ns
Spleen	P<0.05	P<0.05	ns	ns

Control (Cont.); not significant (ns). Group 1 was treated with 10^4^ bacteria and group 2 with 10^5^ bacteria. Each group consisted of 3 cohorts: Control (Cont) and duplicate treatment cohorts, Cage 1 and Cage 2.

1 Three unpaired cohorts in each group were compared using the Kruskal-Wallis test with Dunnett's post test to correct for the number of comparisons. P values less than 0.05 were considered significant.

### P22sTsp retards the motility of *Salmonella*

**Figure 6:**
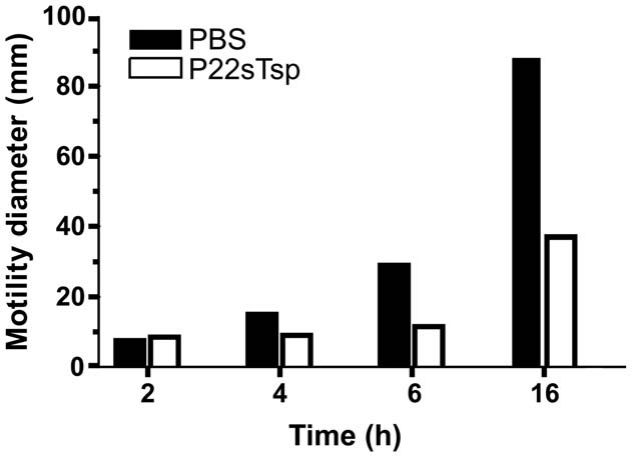
P22sTsp reduces *Salmonella* motility. The spread of *Salmonella* representative of its motility on soft agar plates was measured at different time points and used to plot a graph of motility diameter versus incubation time.

Bacterial motility is implicated in colonization and invasion of host cells by bacteria [42], [43]. To verify if Tsp treatment of *Salmonella* would affect bacterial motility, hence its virulence [44], we performed bacterial motility assays. *Salmonella* cells were applied at the centre of the motility plates with or without P22sTsp throughout the plates and allowed to grow (circles). The diameter of growth was calculated at different time points and used as a measure of motility. The results clearly show that the motility of *Salmonella* is significantly retarded in the presence of P22sTsp (Figure 6).

## Discussion

In this study we showed that oral administration of *Salmonella*-specific shortened tailspike protein (P22sTsp) significantly reduced *Salmonella* infection in chicks. Detailed studies are still required to delineate the mode of action of P22sTsp in reducing *Salmonella* colonization. However, one mechanism could be through the ability of P22sTsp to retard the motility of *Salmonella in vivo,* a possibility supported by our *in vitro* experimental results. This motility-retarding ability of P22sTsps can be attributed to their endorhamnosidase and/or binding activity capable of “modifying” O-antigen, hence, compromising the structure of lipopolysaccharide. This is in line with previous studies showing that (i) motility is a colonization factor for *Salmonella* which can be retarded by altering the structural integrity of lipopolysaccharides, (ii) the lipopolysaccharide O-antigen of a *Salmonella* is a significant factor in gastrointestinal colonization of chicks, and (iii) a *Salmonella* O-antigen-specific antibody inhibited *Salmonella enterica* serovar Typhimurium motility and entry into epithelial cells [42]–[48]. Tsp-mediated bacterial agglutination may also have a role in *in vivo* decolonization, although P22sTsp did not agglutinate *Salmonella in vitro* at physiological temperature under the conditions we tested [49]–[51]. Given that bacterial motility and/or cell surface proteins are often required for infection or retention of bacteria, blocking these cell surface binding sites and/or reducing motility of the bacteria will reduce the infectivity of the organism. It is unlikely that the observed therapeutic effect of Tsp is related to a generalized stimulation of the immune system due to endotoxin contamination of Tsp preparations since phage crude lysates were shown not to be toxic in 7-week old chickens [52] and the 2-day old chicks used in the current study would have immature immune systems [53].

If used in oral therapy, an effective decolonizing agent must be somewhat resistant to proteolysis by GI proteases (pepsin, trypsin, chymotrypsin) and able to withstand the low pH of the gastric juice. *In vitro* digestion studies showed that P22sTsp was sensitive to pepsin and somewhat sensitive to chymotrypsin. To diminish the adverse effect of these enzymes *in vivo*, the administered P22sTsp was formulated in 10% BSA and PBS (a physiological pH buffer), since our *in vitro* digestion experiments indicated that 10% BSA protected P22sTsp from digestion by pepsin and chymotrypsin. With that high of a concentration, BSA presents 1000-fold more potential chymotrypsin and pepsin cleavage sites than P22sTsp (Table S1), and thus becomes the target for chymotrypsin and pepsin digestion. These findings are in agreement with previous ones which showed the co-presence of high concentrations of a protein additive (10% BSA in the present case) protects the therapeutic protein from proteolysis [54]–[57]. Furthermore, the buffering capacity of the formulation (PBS) is also protective as it renders pepsin in the stomach inactive and neutralizes the protein denaturing capability of acidic gastric juice [54], [55], [58], [59]. This may explain why P22sTsp was not digested with the GI fluid which presumably contains pepsin. Formulation of P22sTsp in a higher pH buffer than PBS, e.g., carbonate buffer, or using encapsulation formats should provide P22sTsp with even better protection against the acid-denaturing and peptic environment of the stomach and lead to a more effective therapeutic effect *in vivo* [55], [59], [60].

From the therapeutic point of view in the context of the GI tract and mucosal surfaces in general, Tsps offer several advantages. Tsps (i) can be expressed in *E. coli* in high amounts leading to reduced production costs, (ii) maintain their clonal originality during production, as opposed to intact phages which are prone to frequent mutations, (iii) are stable molecules with significant resistance against GI tract major proteases, (iv) reduce pathogen load without lysing bacteria, hence no harmful bacterial products are released, (v) are target specific and should not disturb the gut's normal microflora, (vi) should show reduced emergence of resistance since Tsps do not threaten bacterial viability, (vii) are expected to be nontoxic, as they are regularly consumed in foods as part of phage (usually more than 10^8^ phages per gram of meat) and (viii) are amenable to protein engineering for improved function. For example, Tsps can be engineered to have improved stability, binding and avidity with novel, narrow or broad specificities. They can also be modified to have additional effector functions by means of fusion to toxins. Other novel functions such as agglutination capabilities at physiological temperatures can be imparted on Tsps through mutations that eliminate the enzymatic activity, but retain the binding activity [25], [33].

While the immediate therapeutic inference of the present findings concerns the treatment of chickens for a more economic production of meat [61]–[64], it has far-reaching implications. One source of human infection with *Salmonella* is through contaminated meat and therefore Tsp treatment of chickens constitutes a prevention-at-source strategy. The concept of the prevention-at-source strategy is also an attractive one in the case of infections caused by human pathogens such as *E. coli* O157:H7, a bacterium which is a commensal in cattle and infects humans through contaminated food and water. This prophylactic treatment is more effective and less costly than the downstream treatment of infected individuals. In this regard, incorporation of Tsps into feed and/or water for animals may prove to be a very practical and cost effective preventative measure for both animals and humans. The Tsp therapy approach can be extended to bacterial infections in humans and “production animals” such as cows, pigs, fish and other livestock in general. In humans, in particular, oral therapy with Tsps, may be useful in combating pathogens such as *Clostridium difficile* or *E. coli* O157:H7 [65].

## Materials and Methods

### Cells, phage and P22sTsp

*S. enterica* serovar Typhimurium (ATCC19585), *S. aureus* (ATCC12598) and P22 phage (ATCC19585-B1) were purchased from the American Type Culture Collection (Manassas, VA). P22sTsp cloning, expression and preliminary characterization is described in a separate publication. Briefly, the truncated form of the P22 tailspike protein (P22sTsp) [29] spanning amino acid residues 109-666 was cloned and expressed in *E. coli*. P22sTsp contained an N-terminal His_6_ tag for purification purposes by immobilized metal affinity chromatography. P22sTsp expression yielded 25–50 mg of purified soluble protein per litre of bacterial culture.

### Growth of bacteria

To prepare cells for micro-agglutination assays, a single *S.* Typhimurium colony from a nutrient agar plate was used to inoculate 15 ml of nutrient broth (NB) and was grown overnight at 37°C with shaking at 250 rpm. The culture was centrifuged, washed with PBS buffer, re-centrifuged and subsequently re-suspended in cold PBS. The cell density was adjusted to an OD_600_ of 1 (1 OD_600_  = 1×10^8^ cells/ml) with PBS. *S. aureus* was grown as described for *S.* Typhimurium, but in brain heart infusion media.

To prepare cells for *in vivo* experiments, a frozen stock of *S.* Typhimurium was streaked onto a xylose lysine deoxycholate (XLD) plate (Oxoid Company, Nepean, ON, Canada) followed by incubation at 37°C for 18–24 h. Three millilitres of overnight culture (in NB) was started from a single colony of *S.* Typhimurium on an XLD plate, and grown at 37°C. This culture would typically have an OD_600_ of approximately 1.4–1.6. A 1∶100 dilution of bacterial cells in 2×10 ml NB was made and the cells were grown at 37°C until they reached an OD_600_ of 0.3–0.5 (∼2–3 h). Cells were centrifuged at 12,000 g and re-suspended in PBS to a final OD_600_ of 1.0. Serial dilutions of the cell culture, on XLD plates, were also performed to confirm the cell density. Cells were immediately used to orally inoculate chicks.

### Micro-agglutination assay

*S.* Typhimurium micro-agglutination assays were performed with P22sTsp or the Se155 IgG control in microtitre plates at 4°C or 42°C [36]. Fifty microlitres of *S.* Typhimurium or control bacteria (*S. aureus*) at an OD_600_ of 1 were added to wells. Subsequently, two-fold serial dilutions of P22sTsp or Se155 IgG in PBS (50 µl) were added to wells. Plates were incubated overnight at 4°C or 42°C. In a micro-agglutination assay, agglutinated cells appear diffused whereas the non-agglutinated ones appear as round dots at the bottom of the wells. The minimum concentration of P22sTsp which resulted in detectable cell agglutination (minimum agglutination concentration) was determined and used as a measure of agglutination potency of P22sTsp.

### Preparation of GI fluid protease solution

To prepare the GI fluid protease solution, four chicks were sacrificed and the GI contents were squeezed out of the GI tract and weighed. The appropriate amount of sterile PBS was added to make a 10-fold dilution. The sample was centrifuged at 1000 g for 15 min at 4°C and 1-ml aliquots of the supernatant was transferred to microfuge tubes and spun at maximum speed to pellet down any remaining debris. The supernatants were frozen at −20°C and stored in small aliquots for future digestion experiments.

### Protease digestion experiments

Freshly-prepared sequencing grade trypsin or chymotrypsin (Hoffmann-La Roche Ltd., Mississauga, ON, Canada) in 1 mM HCl were used for digestion experiments. One microlitre of 0.012 µg/µl trypsin or chymotrypsin was mixed with 8 µl of 0.3 µg/µl P22sTsp in 100 mM Tris-HCl buffer pH 7.8 (trypsin) or in 50 mM Tris-HCl buffer pH 7.8 plus 20 mM CaCl_2_ (chymotrypsin). Reactions were carried out in a total volume of 10 µl for up to 1 h at 37°C and stopped by adding 1 µl of 0.1 µg/µl trypsin/chymotrypsin inhibitor (Sigma-Aldrich Canada Ltd.). Following completion of digestion, samples were mixed with SDS-PAGE loading buffer, incubated for 5 min at 95°C and analyzed by PhastSystem SDS-PAGE according to the manufacturer's instructions (GE Healthcare). Pepsin digestion mixtures contained 8 µl of 0.3 µg/µl P22sTsp, 1 µl of 100 mM HCl and 1 µl of 0.012 µg/µl pepsin (Sigma-Aldrich Canada Ltd.). Reactions were carried out at 37°C for up to 1 h and were subsequently analyzed by SDS-PAGE as described above.

To carry out digestion experiments with the GI fluid protease solution, frozen stocks (see above) were thawed, diluted 10-fold, and 30 µl of the diluted samples was mixed with 50 µg of P22sTsp. Reactions were carried out in PBS buffer in a total volume of 80 µl at 37°C for up to 2 h. The reactions were stopped by immediately boiling the samples for 5 min. In protease-negative experiments, 30 µl of inactivated GI fluid was used (inactivated samples were prepared by heating chick intestinal tract fluid at 95°C for 15 min followed by cooling down on ice). In P22sTsp-negative control experiments, P22sTsp was replaced with a control protein. Following completion of the digestions, aliquots were removed and analyzed by SDS-PAGE.

The sensitivity of P22sTsp to chymotrypsin and pepsin in the presence and absence of 10% (w/v) BSA (Fraction V, Minimum 98%, Sigma) was compared. One microlitre of 0.1 µg/µl chymotrypsin, 40 µl of 0.5 µg/µl P22sTsp and 360 µl of 100 mM Tris-HCl buffer pH 7.8 plus 20 mM CaCl_2_ were combined. For the pepsin digestion experiment, 40 µl of 0.5 µg/µl P22sTsp and 340 µl of PBS were combined. The pH of the reaction mixture was adjusted to 2.0 by adding 20 µl of 1 M HCl followed by the addition of 1 µl of 0.1 µg/µl pepsin. Reactions were carried out in a total volume of 400 µl with or without 10% BSA at 37°C for 1 h and stopped by adding 16 µl of 25x protease inhibitor cocktail (Hoffmann-La Roche Ltd.) to the chymotrypsin digestion or by raising the pH of the reaction mixture to 7–8 with 1 M NaOH for the pepsin digestion. Subsequently, PureProteome™ Nickel Magnetic Beads (Millipore, Billerica, MA) were used to purify His-tagged P22sTsp away from the bulk BSA in the reaction mixtures. The optimal pH for the binding of the nickel beads to the His tag, according to the manufacturer, is 7.5–8.0. After adding 400 µl of the binding buffer (50 mM Tris-HCl/500 mM NaCl/20 mM imidazole pH 7.5) to the 400 µl terminated digestion reactions, including those without 10% BSA, the pH of the reaction mixtures was determined and adjusted to 7.5 to 8.0, if necessary. Fifty microlitres of magnetic beads, equilibrated with the binding buffer, was added to the 800 µl mixture and was incubated for 30 min at 37°C on a desktop rotor. Three consecutive washes were performed with the binding buffer to remove the BSA followed by the elution of nickel-bound P22sTsp with 100 µl of 50 mM Tris-HCl/500 mM NaCl/500 mM imidazole, pH 7.5. Eluted P22sTsp was mixed with SDS-PAGE loading buffer, incubated for 5 min at 95°C and analyzed by SDS-PAGE.

### Motility assay

Motility plates (NB plates/0.4% agar with or without 25 µg/ml filter-sterilized P22sTsp) were made the day before their use and left at room temperature. Purified P22sTsp was buffer-exchanged by size exclusion chromatography (Superdex™ 200 column, GE Healthcare) using PBS as the equilibration buffer and then added to the molten motility media just before pouring them into plates (50°C). To perform motility assays, *Salmonella* cells were grown on NB plates overnight at 37°C (16–18 h). Cells were subsequently suspended in sterile PBS at a cell density of 1 OD_600_. Employing a 10-µl pipettor, 5 µl of the cell suspension was used to inoculate the centre of the motility plates, lightly piercing the surface of the agar plate with the pipettor tip; the plates were left unmoved until the inoculation spots became dry. The plates were then incubated at 37°C. At different time points, the dimensions of the *Salmonella* zones of motility were measured. In control experiments, an equal volume of PBS replaced P22sTsp.

### Animal studies

Leghorn chicks were obtained from the Canadian Food Inspection Agency one day after hatch. The animals were maintained and used in accordance with the recommendations of the Canadian Council on Animal Care Guide to the Care and Use of Experimental Animals. The experimental procedures were approved by the institutional animal care committee (Protocol Number 2004-20). One-day-old chicks were provided with feed and water *ad libitum* and cared for in accordance to the approved guidelines of the Canadian Council for Animal Care. For determining the presence of endogenous *Salmonella*, cloacal swabs were taken from 10% of the chicks, selected at random, with calcium alginate swabs (Fisher Scientific, Ottawa, ON, Canada). XLD plates were streaked with the swabs and incubated at 37°C overnight. The presence of *Salmonella* was identified as black colonies on the red/pink XLD plate.

Infection of 2-day-old chicks was done by oral gavaging the animals with 10^4^–10^7^ *Salmonella*/300 µl PBS. Subsequently, chicks were gavaged a total of 3 doses of P22sTsp (30 µg/300 µl) in 10% BSA or with 10% BSA alone. The 3 oral doses were given at 1 h, 18 h and 42 h (Protocol 1) or at 18 h, 42 h and 66 h (Protocol 2) post-infection and the chicks were subsequently sacrificed at 47 h (Protocol 1) or 71 h (Protocol 2). Following sacrifice, the cecal materials, livers and spleens were collected and processed for determination of *Salmonella* titre as described below.

#### Cecal material

The cecal samples were weighed (≤0.2 g), diluted in PBS (10^−1^ dilution) and vortexed. Further serial dilutions up to 10^−6^ were performed in 200 µl of PBS in 96-well microtitre plates using a multi-channel pipettor. Starting from the highest dilution, 50 µl was plated on XLD plates. The plates were allowed to dry and subsequently incubated at 37°C overnight. The bacterial counts from the serial dilutions were determined the next day.

#### Liver and spleen

To homogenize samples, two Bio 101 ¼” ceramic beads (Qbiogene, Carlsbad, CA) were placed in 2-ml screw cap tubes (Diamed, Mississauga, ON, Canada). Sterile PBS was added to spleen samples (600 µl) or liver samples (800 µl, which were cut from the right front lobe of liver) and kept on ice prior to placing them in the Fastprep Instrument (Qbiogene). Fifty microlitres of the homogenized samples, which resulted in countable colonies, were plated on XLD plates. Plates were incubated overnight at 37°C and the bacterial counts were determined in the morning. Any infected liver with lower than 24 bacteria (2 times the 1/12 dilution factor) or infected spleen with a bacterial count lower than 32 (2 times the 1/16 dilution factor) was below the detection limit of this method.

### Statistics

We assumed a non-Gaussian distribution of the data and therefore used non-parametric tests. To compare 3 or more unpaired groups, the Kruskal-Wallis test with Dunnett's post test was performed to correct for the number of comparisons. Medians of two unpaired groups were compared using the two-tailed non-parametric t-test (Mann-Whitney test). The chi square test was used to compare the pairs. P values less than 0.05 were considered significant. All analyses were performed using GraphPad Prism version 4.02 for Windows (GraphPad Software, San Diego, CA; “www.graphpad.com”).

## Supporting Information

Table S1 Theoretical number of cleavage sites for P22sTsp and BSA.
(0.03 MB DOC)
