# Supplementary material for: Orally Administered P22 Phage Tailspike Protein Reduces Salmonella Colonization in Chickens: Prospects of a Novel Therapy against Bacterial Infections
Source: PLoS One. 2010 Nov 22;5(11):e13904. doi: 10.1371/journal.pone.0013904 (PMC2989905; doi:10.1371/journal.pone.0013904)
Supplement: Table S1 — Theoretical number of cleavage sites for P22sTsp and BSA. (0.03 MB DOC) [file pone.0013904.s001.doc]

**Table S1.** Theoretical number of cleavage sites for P22sTsp and BSA.

| **Enzyme** | **No. of cleavages per protein^1^** | | **Cleavage ratio (BSA/P22sTsp)^2^** |
| --- | --- | --- | --- |
|  | BSA^3^ | P22sTsp |  |
| Chymotrypsin - high specificity | 48 | 46 | 940 |
| [Chymotrypsin - low specificity](http://expasy.org/tools/peptidecutter/peptidecutter_enzymes.html#Ch_lo) | 123 | 103 | 1075 |
| [Pepsin (pH 1.3)](http://expasy.org/tools/peptidecutter/peptidecutter_enzymes.html#Pn1.3) | 162 | 152 | 960 |

^1^No. of cleavages per protein was determined using the [ExPASy](http://expasy.org/) [PeptideCutter](http://expasy.org/tools/peptidecutter/) program (<http://expasy.org/tools/peptidecutter/>).

^2^The ratio of the total number of cleavages for BSA over the total number of cleavages for P22sTsp in the formulation dose used for oral administration (which contained 30 mg BSA and 30 µg P22sTsp).

^3^Mature BSA (Embl accession [Y17769.1](http://www.ncbi.nlm.nih.gov/nuccore/3336841), aa25 – 607) was entered into the program.

Cleavage ratio (BSA/P22sTsp) was calculated as follows:

(No. of cleavages per molecule of BSA) / (No. of cleavages per molecule of P22sTsp) x (molar ratio of BSA to P22sTsp in the formulation dose)

Example for the “Chymotrypsin – high specificity” case:

940 = (48) / (46) x (900)

Thus in this case, for every potential protease cleavage site for P22sTsp there are 940 potential cleavage sites for BSA in the formulation dose used for oral administration.
